# Type 2 diabetes mellitus and non-alcoholic fatty liver disease: a systematic review and meta-analysis

**Published:** 2017

**Authors:** Nasrin Amiri Dash Atan, Mehdi Koushki, Morteza Motedayen, Majid Dousti, Fatemeh Sayehmiri, Reza Vafaee, Mohsen Norouzinia, Reza Gholami

**Affiliations:** 1 *Proteomics Research Center, Faculty of Paramedical Sciences, Shahid Beheshti University of Medical Sciences Tehran, Iran*; 2 *Medicine Faculty, Tehran University of Medical Sciences, Tehran, Iran*; 3 *Department of Cardiology, Faculty of Medicine, Zanjan University of Medical Sciences, Zanjan, Iran*; 4 *Department of Parasitology, Faculty of Medicine, Shiraz University of Medical Sciences, Fars, Iran*; 5 *Student Research Committee, School of Medicine, Shahid Beheshti University of Medical Sciences, Tehran, Iran.*; 6 *Safety promotion and Injury prevention Research Center, Shahid Beheshti University of medical sciences, Tehran, Iran*; 7 *Gastroenterology and Liver Diseases Research Center, Research Institute for Gastroenterology and Liver Diseases, Shahid Beheshti University of Medical Sciences, Tehran, Iran.*; 8 *Basic and Molecular Epidemiology of Gastrointestinal Disorders Research Center, Research Institute for Gastroenterology and Liver Diseases, Shahid Beheshti University of Medical Sciences, Tehran, Iran*

**Keywords:** NAFLD, Type 2 diabetes mellitus, Fatty liver, Meta-analysis

## Abstract

**Aim:**

The aim of this study was the evaluation of the prevalence of NAFLD in patients with type 2 diabetes mellitus.

**Background:**

Non-alcoholic fatty liver disease (NAFLD) is an emerging disease with high prevalence in patients with type 2 diabetes mellitus (T2DM). Many studies have reported the prevalence of NAFLD in type 2 diabetes mellitus patients. However, these results are inconsistent.

**Methods:**

A Literature search was conducted in PubMed, Scopus, web of science and Science Direct from 2005 to August 2017. The necessary information was extracted. Heterogeneity was evaluated using I^2^ statistic. Meta-regression analyses were performed to the estimation of the relationship between the year of study and sample size with the prevalence of NAFLD. Publication bias was assessed by both Begg rank correlation and Egger tests. Subgroup analysis was performed for identification of sources heterogeneity.

**Results:**

Seventeen studies involving 10897 type 2 diabetes mellitus patients with NAFLD were included in this meta-analysis. The overall prevalence of NAFLD in type 2 diabetes mellitus patients by random effects models was 54% (95% CI, 45%- 64%). There is a significant heterogeneity across studies with (I^2^= 99%, p> 0.01). The funnel plot as graphically and Begg and Egger as statistically showed no publication bias among studies. Subgroup analysis indicated that the prevalence of NAFLD in type 2 diabetes mellitus patients differed in predictive factors such as lipid profile, BMI, HbA1c, AST, and ALT. This finding in spite of heterogeneity of documents is corresponding to the positive correlation between NAFLD and type 2 diabetes mellitus.

**Conclusion:**

The findings indicated that the overall prevalence of NAFLD among type 2 diabetes mellitus patients is significantly higher. It can be concluded that type 2 diabetes mellitus patients should be managed to prevent NAFLD.

## Introduction

 Non-alcoholic fatty liver disease (NAFLD) as one of the most common liver diseases is emerging as a public health problem issue worldwide(1). The disease occurs when fat forms more than 10 to 5% of the liver's weight. The disease involves a wide range of liver diseases but occurs in people who either do not drink alcohol or only use moderate amounts of alcohol(2). NAFLD incorporates histologically and clinically different non-alcoholic entities; fatty liver (NAFL, steatosis hepatic) and steatohepatitis (NASH characterized by hepatocyte ballooning and lobular inflammation ± fibrosis) might progress to cirrhosis and rarely to hepatocellular cancer(3). Obesity and physical inactivity are interlinked risk factors for the development of diabetes NAFLD(4). Diagnosis of NAFLD based on liver biopsy, ultrasound evidence of bright liver, and posterior attenuation in people without alcohol or low alcohol consumption(5). In an extensive study about the prevalence of NAFLD in type 2 diabetes mellitus in Italy, the results indicated the 70% prevalence of NAFLD in type 2 diabetes mellitus patients. Also recently, several studies have been reported that the prevalence of NAFLD in type 2 diabetes mellitus patients ranges broadly between 34% - 94%(6). The prevalence of NAFLD is increasing and approximately 34%-46% of obese adult in developed countries have NAFLD(7). It is well known that the prevalence of NAFLD associated with several risk factors such as obesity, metabolic syndrome, insulin resistance and type 2 diabetes(8, 9). There is a strong association between NAFLD and diabetes risk. An individual's risk of developing diabetes is increased approximately 5-fold if they have NAFLD(10, 11). The association between NAFLD and type 2 diabetes could be explained by the insulin resistance, dyslipidemia and hepatic triglyceride (TG) accumulation in NAFLD and defective B-cell in type 2 diabetes mellitus (9). Compared to healthy populations, type 2 diabetes mellitus patients show increased risk for catching of advanced liver disease including fibrosis, cirrhosis and hepatocellular carcinoma(12). In this regard, an accurate estimate of NAFLD prevalence in type 2 diabetes mellitus patients is important. However, there was high inconsistency across the results of studies in the estimated prevalence of NAFLD in type 2 diabetes mellitus patients. Therefore, the aim of this meta- analysis is a determination of the overall prevalence of NAFLD and its predictive variables in type 2 diabetes mellitus patients. 

## Methods

**Search strategy**

This meta-analysis was carried out with literature search of the electronic databases of PubMed, Scopus, web of science, ScienceDirect and Embase from 2005 to August 2017. Key words: (“Non-alcoholic Fatty Liver disease” AND Diabetes Mellitus, type 2), (NAFLD AND T2DM), (NAFLD, Diabetes Mellitus) were used to identify relevant studies. Meanwhile, the reference lists of full articles were reviewed. 

**Study inclusion/ exclusion criteria**

The studies were included if they had the following inclusion criteria: 1) studies published in English language, 2) reported the prevalence of NAFLD in T2DM patients and 3) provided the necessary information of relevant studies and also studies exclude if they had following criteria: reviews, case-reports, comments, brief reports abstracts or book chapters because of limited data.

**Data extraction**

All studies were assessed independently by two reviewers and extracted data from included studies in this meta-analysis. The extracted data as follows: first author, publication year, sample size, mean age of type 2 diabetes mellitus patients, the prevalence of NAFLD (%)in type 2 diabetes mellitus and laboratory parameters including mean (SD) total cholesterol, HGL, LDL, HbA1c, ALT and AST.

**Quality assessment**

In this meta-analysis, the PRISMA was used to evaluate the quality of eligible studies. PRISMA focuses on ways in which authors can ensure the transparent and complete reporting of systematic reviews and meta-analysis. It does not address directly or in a detailed manner the conduct of systematic review and meta-analysis. The PRISMA statement consists of 27-item checklist. The checklist includes items seem to be essential for transparent reporting of systematic review and meta-analysis.

**Statistical analysis**

Statistical analyses for this meta-analysis were performed using STATA version 14.0 (STATA Corporation, College Station, TX, USA). The pooled prevalence of NAFLD in type 2 diabetes mellitus patients was performed as a percentage with corresponding 95% CI by a random- effects model when significant heterogeneity was observed (p<0.1 and I^2^>50%). The association between the prevalence of NAFLD in type 2 diabetes mellitus patients with publication year across studies was performed by meta-regression analyses. Publication bias was assessed using both Begg rank correlation test as statistically and funnel plot as graphically. Subgroup analysis were performed to identify the possible sources of heterogeneity. *P-value* of <0.05 was considered significant.

## Results

**Selection of studies**

**Figure 1:**
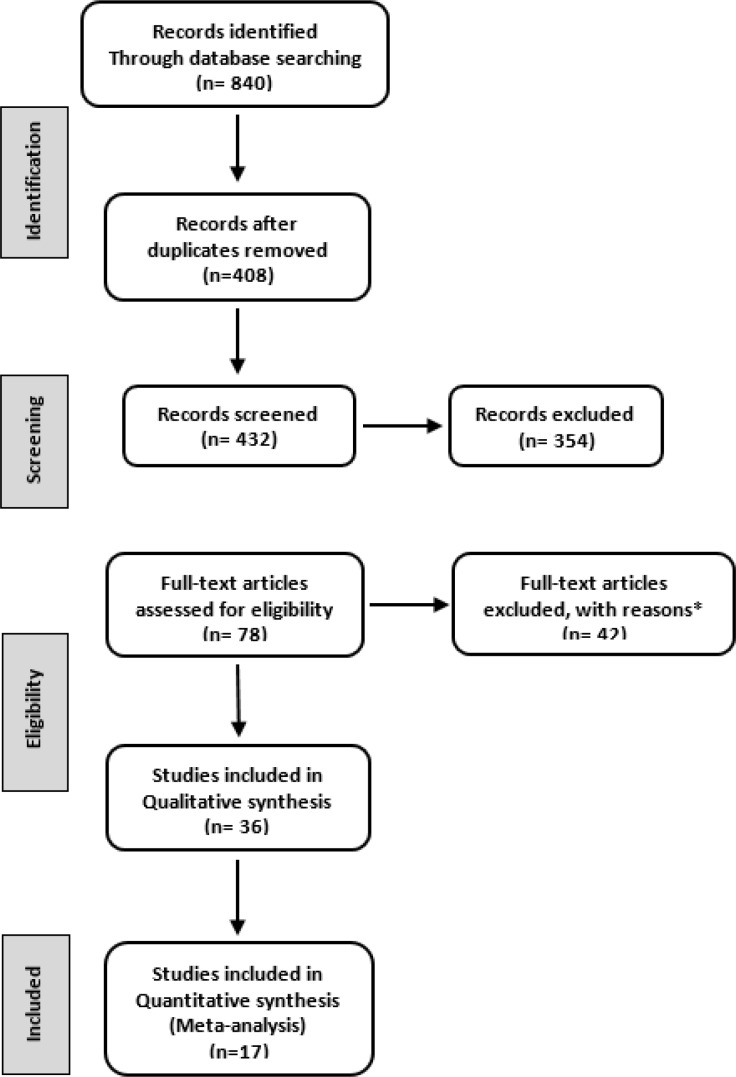
The flow chart of studies selection

Initially, a total of 840 articles were searched in this study. Of these 408 articles were excluded due to duplicate data, 354 articles were excluded for not reporting the prevalence of NAFLD in type 2 diabetes mellitus patients, 42 articles excluded for not having available data, 19 articles were excluded for not being review article, abstract or a brief report. Finally, 17 eligible articles were selected in this meta-analysis (Figure 1).

**Study characteristics**

We identified 17 articles that reported the prevalence of NAFLD in type 2 diabetes mellitus patients. Table 1 lists the studies used in this meta-analysis along with baseline characteristics. A total of 10897 individuals participated in 17 articles.

**The prevalence of NAFLD in patients with type 2 diabetes mellitus**

**Figure 2:**
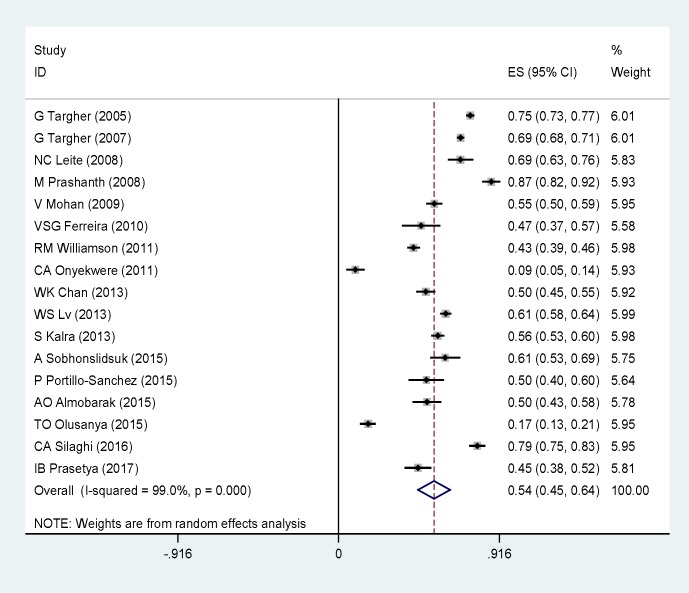
The forest plot of NAFLD prevalence in type 2 diabetes patients. Square represents effect estimate of individual studies with more than 95 % confidence intervals with the size of squares proportional to the weight assigned to the study in the meta-analysis. In this chart, studies are stored in order of the year of publication and author’s names, based on a random effects model

Figure 2 presents the results of the overall prevalence of NAFLD in type 2 diabetes mellitus patients that calculated as percentage with corresponding 95% CI by a random effects model. The pooled prevalence of NAFLD in type 2 diabetes mellitus patients was 54% (95% CI 45%- 64%). The prevalence of NAFLD as outcome was significantly high in type 2 diabetes mellitus. There was statistically significant heterogeneity between studies (p< 0.01, I^2^= 99%). To explore reasons for heterogeneity, we conducted subgroup analysis. We found significant heterogeneity for total cholesterol and TG that were (I^2^= 98%) and (I^2^= 97%) respectively. The pooled prevalence of NAFLD in type 2 diabetes mellitus patients with high BMI was 29% (28.09 – 29.91), (I^2^= 98.9%). 

**Figure 3.**
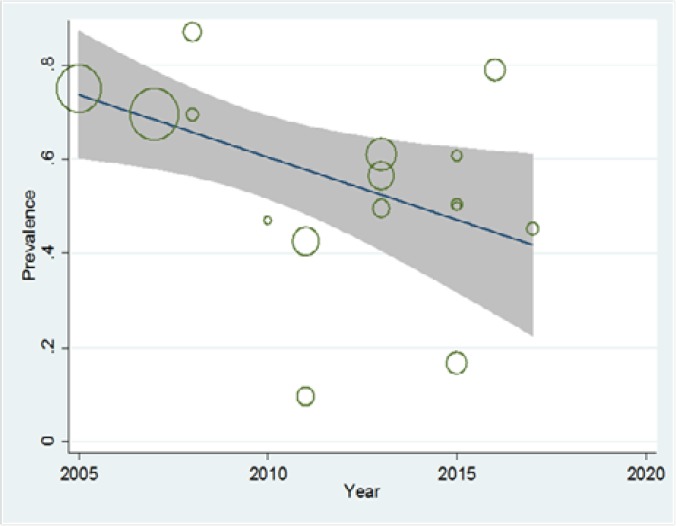
The prevalence of NAFLD in type 2 diabetes patients based on year of study. The fitted line shows meta-regression line

**Figure 4:**
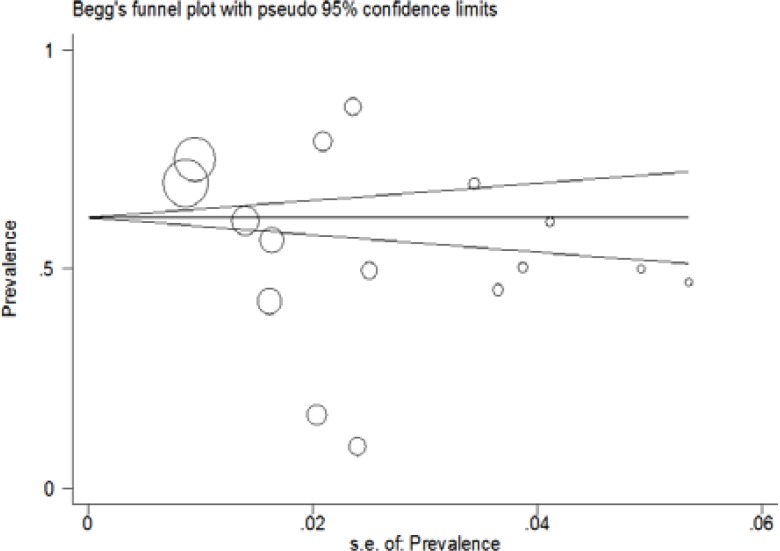
Funnel Plot to assess Publication Bias. The diameter of each circle represents the weight in the meta-analysis

The subgroup analyses indicated that the prevalence of NAFLD in type 2 diabetes mellitus differed to assess total cholesterol, TG, BMI, ALT, and AST. Table 2 shows the results of subgroup analyses. Visual inspection of the funnel plot and the Begg rank correlation method (p= 0.11) (Fig 3) have not shown any publication bias. We performed meta-regression test for the association between the prevalence of NAFLD in type 2 diabetes mellitus patients with publication year of articles that no significant change observed (p= 0.16) (Fig 4). Taken together, these findings suggest that the prevalence of NAFLD in type 2 diabetes mellitus patients is high. Therefore, the early diagnosis of NAFLD is essential for NAFLD management in type 2 diabetes mellitus patients.

**Table 1:** The studies characteristics included in this meta-analysis. TG: Triglyceride, HLD: High lipoprotein density, LDL: Low density lipoprotein, BMI: Body mass index

ALT (M±SD)	AST (M±SD)	BMI (M±SD)	HbA1C (M±SD)	LDL (M±SD)	HDL (M±SD)	TG (M±SD)	Total cholesterol (M±SD)	Prevalence (%)	Age (M±SD)	Sample size	Country	Year	First author (references)
-	-	31.3±5.7	7.2±1.1	84.6±26.5	48±13.3	147.7±80.1	161.9±31.6	42.6	68.9±4.2	939	UK	2011	Williamson (13)
25±3	23±3	28.3±4	7.3±1.1	131.4±15.6	52.3±15.6	149.5±89		69.5	65±6	2839	Italy	2007	Targher (14)
20.3±26	15.6±11.3	33.9±8.3		103.5±57	36.6±15.1	100.6±61.3	170.7±100.5	9.5	52.5±10.9	150	Nigeria	2011	Onyekwere (15)
21.8±10.3	18.9±5.7	29.5±4.8	8.8±2.7	122.3±41.3	48.4±10.4	164.1±84.4	204.7±49.5	47	57.3±10.3	87	Brazil	2010	Ferreira (16)
33±14	26±12	29±4	7.2±0.9	127.5±19.5	48.8±15.6	144.2±53.4	-	75	66±4	2103	Italy	2005	Targher (17)
47.7±21.9	27.3±12.3	27.6±4.1	7.2±1.3	-	-	161.5±75.7	183.9±38	60.7	60.47±9.8	141	Thailand	2015	Sobhonslids (18)
37±18	23±12	29.7±7.9	8.31±1.7	82.7±68.2	46.8±14.4	133.5±106.8	165.8±62.4	49.6	60.7±11.2	399	Malaysia	2013	Chan (19)
-	-	-	7.9	126.75	41.3±16.7	-	-	79	55.7±8.9	381	Romania	2016	Silaghi (20)
39	20	31.3±5.4	7.3	130±44.8	43.3±12.5	113.92	202.8±52.7	69.4	55.6±7.1	180	Brazil	2008	Leite (21)
28.6±19.2	23.7±11.1	25.2±4	6.7±1.6	-	-	128	185±36	54.5	46±12	541	India	2009	Mohan (22)
23.7±12.08	26.97±8.1	26.6±3.85	7.69±1.5	110.3±41.6	46.2±9.4	173.5±68.8	190.6±44	87	54.2±9.2	204	India	2008	Prashanth (23)
23	34.7±5.3	-	7	91±34	40±12	142	164±40	5	58±8	103	USA	2015	Portillo-Sanchez (24)
25.72±13.1	22.34±9	27.5±3.37	8.9±2.5	115±32	45.6±11	188.7±127.3	196.6±47.6	61	62.3±12.5	1217	China	2013	Lv (25)
-	-	-	-	-	-	-	-	45.2	-	186	Indonesia	2017	Prasetya (26)
-	-	-	-	-	-	-	-	50.3	-	167	Sudan	2015	Almobarak (27)
-	-	-	-	-	-	-	-	16.7	53.2±8.6	336	Nigeria	2015	Olusanya (28)
55.6±39.8	54.8±36.1	-	-	-	-	-	-	56.5	52.2±10.8	924	India	2013	Kalra (29)

AST: Aspartate aminotransferase, ALT: Alanine aminotransferase

**Table 2:** The result of subgroup analysis.

Subgroup	Study number	I^2^	SEM(95%CI)
Total Prevalence of NAFLD in People With Type 2 Diabetes	17	99	54(45-64)
Cholesterol	10	98/4	182.63(171.46-193.79)
Triglycerides	9	97	151.29(140.25 -162.33)
HDL	11	98	45.31(43.10 -47.53)
LDL	11	99.7	111.49(102.27-120.72)
HbA1C	12	98.8	7.60(7.35-7.84)
BMI	11	98.9	29 (28.09 -29.91)
AST	11	100	24.59(15.54-33.64)
ALT	10	99.4	31.77(28.09-35.44)

## Discussion

In this systematic review and meta-analysis, prevalence of NAFLD in type 2 diabetes mellitus patients is investigated. A significant increase in the prevalence of NAFLD in type 2 diabetes mellitus patients was observed. The pooled prevalence was 54% (95% CI 45% - 64%). Whereas, in eligible studies involved in this meta-analysis, the prevalence of NAFLD in type 2 diabetes mellitus patients ranged from 5% (24) to 87%(23). Here, the high prevalence of NAFLD in type 2 diabetes mellitus patients indicated the importance of management and early evaluation of NAFLD in type 2 diabetes mellitus patients. The heterogeneity was significant across studies; therefore, subgroup analysis was performed. Subgroup analysis indicated that the prevalence of NAFLD in type 2 diabetes mellitus patients differed by total cholesterol, TG, BMI, HbA_1_c and HDL, LDL, AST, and ALT. The prevalence of NAFLD in type 2 diabetes mellitus patients was 182.63%, 151.29%, 45.31%, 111.49%, 29%, 24.59% and 31.77% for total cholesterol, triglyceride (TG), HDL, LDL, BMI, AST and ALT, respectively. In a recent study, Yi et al. demonstrated that the prevalence of NAFLD in men is higher than females in type 2 diabetes mellitus patients(30). This gender difference in the prevalence of NAFLD could be attributed to the lipid value in female(31). The triglyceride/high density lipoprotein cholesterol (TG/HDLC) ratio seems to be higher in men than women(30). Obesity is one of the most important factors involved in NAFLD that also has been reported in various other studies(32). In our meta-analysis, high BMI in type 2 diabetes mellitus patients is associated with NAFLD. Bhatt .K et al. found that BMI was significantly higher in patients with NAFLD than a control group without NAFLD(33). We observed that transaminases (ALT and AST) levels in the prevalence of NAFLD in type 2 diabetes mellitus patients were not statistically high in subgroup analysis versus the pooled prevalence of NAFLD. However, several studies have shown that there is no correlation between transaminases levels and the prevalence of NAFLD in type 2 diabetes mellitus patients(32). Whereas, Lu et al. reported the prevalence of NAFLD in type 2 diabetes mellitus patients was significantly associated with elevated ALT(34). In this meta-analysis, we found that the subgroup analysis of HbA_1_c in the prevalence of NAFLD is lower than the pooled prevalence of NAFLD in type 2 diabetes mellitus patients, as, it is suggested that there is an unusual relationship between HbA_1_c and NAFLD. 

This systematic review and meta-analysis have several strengths. This review obtained the most comprehensive data from relevant studies pertaining to the prevalence of NAFLD in type 2 diabetes mellitus patients. 

Our search strategy was very detailed. Statistical tests showed no evidence of publication bias in the analyses. Subgroup analyses was conducted to explore possible sources of heterogeneity. 

In the other hand, this systematic review and meta-analysis also have several limitations: first, owing to the small number of studies for a specific outcome, we were not able to better deduce from this studies. Second, we pooled prevalence from studies that appeared to be consisted of adults with mean ages from 46 to 69, whereas, in this meta-analysis adolescent remains unclear. Third, there was substantial heterogeneity between eligible studies. Given the limitations, the finding should be interpreted with caution.

It can be concluded that overall prevalence of NAFLD among type 2 diabetes mellitus patients is significantly higher than the other types of diabetes mellitus. It implies more cares in the type 2 diabetes mellitus patients to prevent NAFLD.

## Conflict of interests

The authors declare that they have no conflict of interest.
